# A standard enteral formula versus an iso-caloric lower carbohydrate/high fat enteral formula in the hospital management of adolescent and young adults admitted with anorexia nervosa: a randomised controlled trial

**DOI:** 10.1186/s40337-021-00513-6

**Published:** 2021-12-11

**Authors:** Elizabeth Kumiko Parker, Victoria Flood, Mark Halaki, Christine Wearne, Gail Anderson, Linette Gomes, Simon Clarke, Frances Wilson, Janice Russell, Elizabeth Frig, Michael Kohn

**Affiliations:** 1 Department of Dietetics and Nutrition, Westmead Hospital, PO Box 533, Wentworthville, NSW 2145 Australia; 2 Sydney School of Health Sciences, Faculty of Medicine and Health, The University of Sydney, Sydney, NSW 2006 Australia; 3 Western Sydney Local Health District, Westmead, NSW 2145 Australia; 4 Department of Medical Psychology, Westmead Hospital, Westmead, NSW 2145 Australia; 5 Department of Adolescent and Young Adult Medicine, Westmead Hospital, Westmead, NSW 2145 Australia; 6 Centre for Research Into AdolescentS’ Health (CRASH), Westmead Hospital, Westmead, NSW 2145 Australia; 7 Sydney School of Medicine, Faculty of Medicine and Health, The University of Sydney, Sydney, NSW 2006 Australia; 8 Department of Psychiatry, Westmead Hospital, Westmead, NSW 2145 Australia; 9 NSW Statewide Eating Disorder Service, Peter Beumont Unit, Professor Marie Bashir Centre, Royal Prince Alfred Hospital, Camperdown, NSW 2050 Australia; 10 Department of Nutrition and Dietetics, Royal Prince Alfred Hospital, Camperdown, NSW 2050 Australia

**Keywords:** Anorexia nervosa, Enteral nutrition, Refeeding syndrome, Hypophosphatemia, Carbohydrate

## Abstract

**Background:**

The nutritional rehabilitation of malnourished patients hospitalised with anorexia nervosa is essential. The provision of adequate nutrition must occur, while simultaneously, minimising the risk of refeeding complications, such as electrolyte, metabolic, and organ dysfunction. The aim of this study was to compare the efficacy and safety of an iso-caloric lower carbohydrate/high fat enteral formula (28% carbohydrate, 56% fat) against a standard enteral formula (54% carbohydrate, 29% fat).

**Methods:**

Patients (aged 15–25 years) hospitalised with anorexia nervosa were recruited into this double blinded randomised controlled trial. An interim analysis was completed at midpoint, when 24 participants, mean age 17.5 years (± 1.1), had been randomly allocated to lower carbohydrate/high fat (n = 14) or standard (n = 10) feeds.

**Results:**

At baseline, there was no significant difference in degree of malnutrition, medical instability, history of purging or serum phosphate levels between the two treatment arms. A significantly lower rate of hypophosphatemia developed in patients who received the lower carbohydrate/high fat formula compared to standard formula (5/14 vs 9/10, *p* = 0.013). The serum phosphate level decreased in both feeds, however it decreased to a larger extent in the standard feed compared to the lower carbohydrate/high fat feed (standard feed 1.11 ± 0.13 mmol/L at baseline vs 0.88 ± 0.12 mmol/L at week 1; lower carbohydrate/high fat feed 1.18 ± 0.19 mmol/L at baseline vs 1.06 ± 0.15 mmol/L at week 1). Overall, serum phosphate levels were significantly higher in the lower carbohydrate/high fat feed compared with standard feed treatment arm at Week 1 (1.06 ± 0.15 mmol/L vs 0.88 ± 0.12 mmol/L, *p* < 0.001). There was no significant difference in weight gain, number of days to reach medical stability, incidence of hypoglycaemia, or hospital length of stay.

**Conclusions:**

The results of this study indicate that enteral nutrition provided to hospitalised malnourished young people with anorexia nervosa using a lower carbohydrate/high fat formula (28% carbohydrate, 56% fat) seems to provide protection from hypophosphatemia in the first week compared to when using a standard enteral formula. Further research may be required to confirm this finding in other malnourished populations.

*Trial Registration*: ANZCTR, ACTRN12617000342314. Registered 3 March 2017, http://anzctr.org.au/Trial/Registration/TrialReview.aspx?ACTRN=12617000342314

**Supplementary Information:**

The online version contains supplementary material available at 10.1186/s40337-021-00513-6.

## Plain English Summary

Patients hospitalised with anorexia nervosa require nutrition support as part of their treatment, whilst refeeding complications are prevented. Of particular concern, is the reintroduction of carbohydrate to malnourished patients, which has been proposed to cause a surge in insulin levels and disturbance in electrolytes, particularly a decrease in blood phosphate levels. This double-blinded randomised controlled trial measured the occurrence of low phosphate blood levels and other refeeding complications, in adolescent and young adult patients hospitalised with anorexia nervosa. These patients were provided either a lower carbohydrate/high fat feed (28% carbohydrate, 56% fat) or a standard enteral feed (54% carbohydrate, 29% fat). Fewer patients in the lower carbohydrate/high fat feed group (5/14) than standard feed group (9/10) developed a low phosphate level. There was no significant difference in weight gain, number of days to reach medical stability, occurrence of hypoglycaemia, or hospital length of stay.

**Supplementary Information:**

The online version contains supplementary material available at 10.1186/s40337-021-00513-6.

## Introduction

Patients hospitalised with anorexia nervosa (AN) require nutritional rehabilitation to (1) achieve medical stability, (2) restore positive energy balance (3) commence weight restoration, and (4) reverse the medical complications associated with malnutrition [1, 2]. Malnourished patients, such as those with AN, are considered at increased risk of developing refeeding complications, such as the refeeding syndrome. While consensus is lacking on a definition of refeeding syndrome, it is generally described as the occurrence of electrolyte and fluid shifts which can occur when a person in a state of starvation undergoes nutrition repletion, leading to organ dysfunction and possible sudden death [1, 3].

A range of international refeeding guidelines provide consensus-based recommendations for initial energy intakes in malnourished patients at risk of developing refeeding complications. The United Kingdom based National Institute for Health and Care Excellence (NICE) guidelines initially published in 2006 and updated in 2017, recommend providing low energy intakes (5–10 kcal/kg/day) to prevent refeeding complications, and aim to increase slowly to meet nutrition needs by 4–7 days [4]. In the USA, the American Society for Parenteral and Enteral Nutrition (ASPEN) guidelines recommend initiating patients on 10–20 kcal/kg for the first 24 h and increasing by 33% of goal energy intake every 1–2 days [5]. These guidelines also suggest delaying increases in energy intakes in the presence of electrolyte derangement. The caloric prescription from these guidelines has been identified as leading to an ‘underfeeding syndrome’ [6], whereby patients are provided with energy intakes below basal energy requirements, resulting in poor weight gain and even weight loss in an already malnourished patient group.

A growing body of evidence supports higher energy intakes in adolescent patients hospitalised with AN, ranging from 1200 to 2400 kcal/day commencing at admission [7]. However, evidence for the adult population is less robust; the fewer studies that exist suggest starting malnourished adults on energy intakes of 1200–1500 kcal/day [8–10].

Concern for the development of refeeding complications is increased when nutrition is commenced at a high caloric rate, the patient is severely malnourished (BMI < 14 kg/m^2^) or when the carbohydrate intake is high [4, 11, 12]. During starvation the body is in a catabolic state. An “adaptive” shift from carbohydrate to fat and protein utilization occurs, which alters the body’s insulin response [13]. Once adequate nutrition is reintroduced, the body returns to an anabolic state, and switches back from fat and protein utilisation to carbohydrate utilisation as the primary energy macronutrient. A sudden reintroduction of carbohydrate during this time is postulated to lead to a surge in insulin levels, which drives electrolytes such as phosphate into the cells, resulting in electrolyte derangements [14]. Hypophosphatemia, is considered a marker for refeeding complications, and serum phosphate levels are recommended to be maintained above 1.0 mmol/L during nutritional rehabilitation [15–18].

Strategies to avoid refeeding complications have been suggested, such as continuous delivery of nutrients (e.g. nasogastric feeding) with less than 40% of energy from carbohydrate [13]. Standard enteral formula usually contain > 50% energy from carbohydrate. A recent study published by Yamazaki T et al. [19], retrospectively reviewed 188 patients hospitalised with AN (mean age 28.77 ± 12.22 years) and found a diet high in carbohydrate (> 58.4%) was significantly associated with the occurrence of refeeding hypophosphatemia (Adjusted OR 5.37, 95% CI 1.60,18.1, *p* = 0.007)[19].

Not all studies of higher energy intakes (initiating ≥ 1200 kcal/day) have specified the macronutrient composition provided to patients during treatment [8, 17, 20, 21]. Studies which have specified the macronutrient content and utilised nasogastric feeding have ranged from starting calorie levels of 1500–2400 kcal/day and 44 to ≤ 50% carbohydrate [10, 18, 22], whereas oral meal based programs have ranged from 1200 to 2000 kcal/day and 35–60% carbohydrate [15, 23–28].

This study compares refeeding treatment outcomes of a lower carbohydrate enteral formula (less than 40% energy from carbohydrate) against a standard enteral formula (54% energy from carbohydrate), in adolescent and young adult patients (aged 15–25 years), hospitalised with AN. In addition, the lower carbohydrate formula is also higher in fat compared to the standard enteral formula (56% energy from fat vs 29% energy from fat, respectively).

Our hypothesis is that compared with malnourished patients with AN provided with a standard higher carbohydrate enteral formula, malnourished patients with AN who are provided with a lower carbohydrate/high fat enteral formula will (1) have lower rates of hypophosphatemia, and (2) have less electrolyte disturbance, and thus safely reach the initial goal rate of feeding (1890 kcal Day 1) sooner. This will affect initial total energy intake, rate of weight gain, number of days to reach medical stability and length of hospital stay.

## Methods

### Study design

This double blinded randomised controlled trial was conducted in Sydney, Australia, from November 2017 to December 2018. A detailed research protocol has been published elsewhere [29]. This study received ethics approval from the Western Sydney Local Health District Human Research Ethics Committee (HREC/16/WMEAD/390) and site specific approvals from the Research Governance Offices of Western Sydney Local Health District (SSA/16/WMEAD/433) and Sydney Local Health District (SSA/17/RPAH/435). This study followed the Consolidated Standards of Reporting Trials (CONSORT) reporting guideline.

### Participants and recruitment

**Fig. 1:**
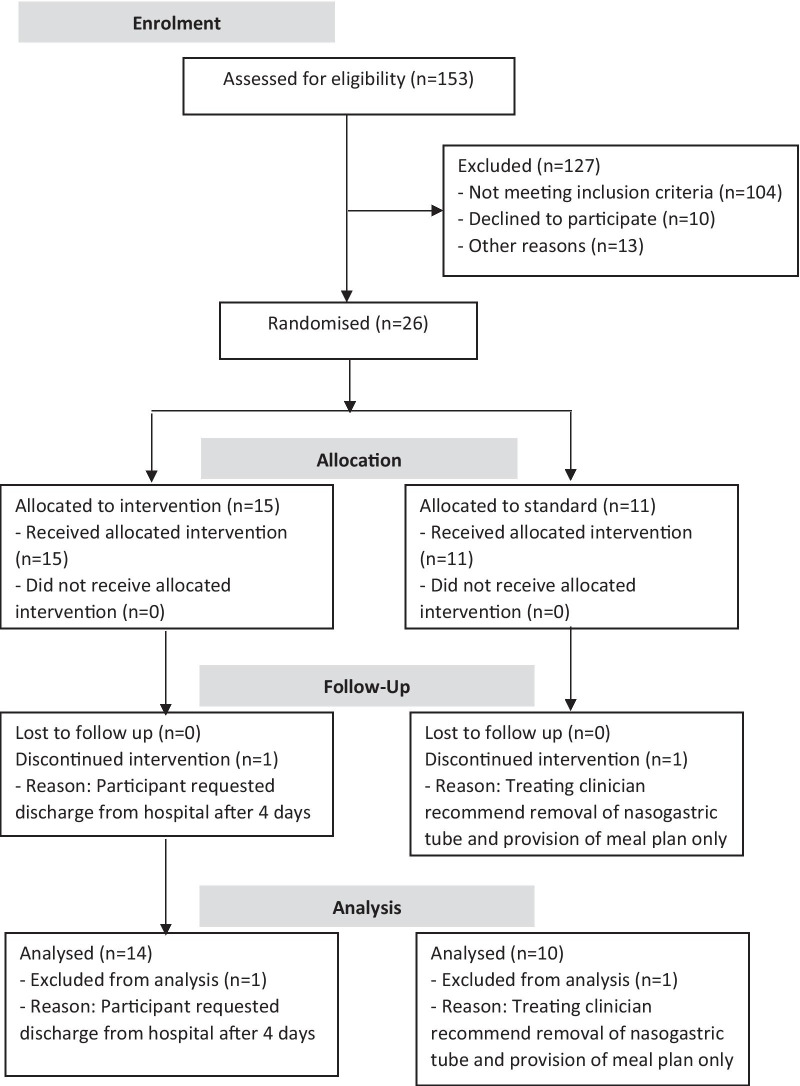
Consortium diagram of patient flow

Recruitment of participants was open at two public hospitals in New South Wales, Australia, with specialised inpatient eating disorder treatment services. Inclusion criteria specified adolescent and young adult patients (aged 15–25 years) hospitalised with AN (assessed by the treating medical officer using DSM-5 criteria [30]) who received nasogastric tube feeding. Exclusion criteria included patients transferred from another treatment facility, where they had already received nasogastric feeding and/or prophylactic phosphate replacement. Figure 1 describes the enrolment of participants into the trial.

Participants were invited to participate shortly after admission to hospital, and were approached after the treating team had made the decision the patient required nasogastric feeding, independent to the study. Written informed consent was obtained from all participants, and from guardians for participants aged less than 18 years.

### Sample size

The sample size calculation of 48 participants (24 in each arm) was based on the primary outcome measure, incidence of hypophosphatemia, using a dichotomous endpoint two independent study sample, incidence of 45% hypophosphatemia [26] in the standard feed arm and 10% hypophosphatemia in the trial feed arm, 80% power, alpha 0.05.

An interim analysis was planned and completed at midpoint of recruitment, with the plan to cease recruitment early if a significant finding in the primary outcome measure of incidence of hypophosphatemia was found.

### Randomisation

Random allocation was concealed by sequentially numbered, sealed opaque envelopes containing the feed allocation (Feed A vs Feed B), which was determined by a computer generated random number, stratified by gender. An external investigator (MH) generated the allocation sequence, and ward staff enrolled participants.

### Blinding

The Department of Dietetics & Nutrition at the study hospital assisted in assigning participants to the randomised interventions and facilitated the blinding of participants and treating team during the intervention. The standard formula and lower carbohydrate/high fat formula were both decanted into generic containers and labelled either FEED A or FEED B. A record logbook of each participant and the enteral feed provided and the allocation to FEED A or FEED B was maintained and stored in a locked cabinet for quality assurance and auditing purposes.

### Intervention group

The trial enteral feed was a lower carbohydrate/high fat formula (Abbott Nutrition™), providing 1.5 kcal/mL and 28% energy from carbohydrate, 56% energy from fat, 17% energy from protein.

### Control group

Participants randomised to the standard enteral feed received an isocaloric formula (Abbott Nutrition™), providing 1.5 kcal/mL, and 54% energy from carbohydrate, 29% energy from fat, 17% energy from protein.

Both groups were provided nutrition in a standardised and similar manner. Initially nasogastric tube feeds were commenced at 35 mL/h for 12 h. After 12 h, feeds were increased to goal rate 70 mL/h continuous infusion (total 1260 mL = 1890 kcal on Day 1), if electrolytes potassium and magnesium were within the normal reference range and serum phosphate was > 1.0 mmol/L, otherwise feed remained at 35 mL/h (total 840 mL = 1260 kcal on Day 1) until electrolytes had normalised.

Once patients were assessed as medically stable, defined as heart rate > 50 bpm, temperature > 35.5 °C, blood pressure > 80/50 mm Hg, postural hypotension < 20 mm Hg, nasogastric feeds were reduced to cyclic nocturnal at a rate of 70 mL/h over 10 h (2000–0600 h) and oral intake was introduced using standardised meal plans (energy content: 1800 kcal, 2300 kcal, 2800 kcal, 3300 kcal, 3800 kcal; macronutrient content: 47–57% carbohydrate, 30–38% fat, 13–15% protein) (Additional file 1: Table S1). Changes to the feeding regime were adjusted as per standard care, following a multidisciplinary team review three times/week, which aimed to support the development of anabolism and weight gain of at least 1 kg per week. Oral intake (main meals and mid meals) was supervised by a ward nurse and recorded on a daily food chart, and participants unable to finish the prescribed meal plan were provided with a nutrition supplement drink as a meal replacement.

Participants received a daily oral multivitamin. Participants did not receive prophylactic phosphate supplementation if baseline serum phosphate was > 1.0 mmol/L. Participants did receive 1 g oral phosphate supplementation prior to commencing nasogastric feeds if baseline serum phosphate levels were ≤ 1.0 mmol/L, and were provided with phosphate supplementation if serum phosphate levels were ≤ 1.0 mmol/L during nutritional rehabilitation. Participants did not receive prophylactic magnesium or potassium supplementation prior to commencing nasogastric feeds, however oral supplementation was provided if clinically indicated.

### Primary and secondary outcomes

The primary outcome measure was incidence of hypophosphatemia. Secondary outcome measures were change in weight, total energy intake and macronutrient content, length of hospital stay, hypokalaemia, hypomagnesemia, hypoglycaemia, development of peripheral oedema, clinical refeeding syndrome, thiamine (Vitamin B1), admission to ICU, days required to reach medical stability.

Measurements of primary and secondary outcomes occurred during the first 3 weeks of hospital admission.

### Nutrition assessment

Nutrition assessment was completed by an accredited practising dietitian, using a validated nutrition assessment tool the Subjective Global Assessment (SGA) [31], to assess change in weight, change in oral intake, nutrition impact symptoms, change in functional capacity, and the presence of muscle wasting, loss of subcutaneous fat stores and oedema. The nutritional status of participants was scored using an SGA rating of A (well nourished), B (mildly / moderately malnourished), or C (severely malnourished). In addition, degree of malnutrition was also assessed in participants using criteria defined in the Position Paper of the Society for Adolescent Health and Medicine [32], using percentage median Body Mass Index (%mBMI), calculated from the 50th percentile for age and sex [33]. Degree of malnutrition using %mBMI was categorised as mild malnutrition (80–90%mBMI), moderate malnutrition (70–79%mBMI), or severe malnutrition (< 70%mBMI) [32].

Nutritional intake of participants was assessed using recorded food charts and recorded administered enteral feed volumes.

Change in body weight was assessed at routine weight checks, minimum 3 days/week, using the ward scales recorded to the nearest 0.1 kg. These were early morning weights taken after voiding. Body mass index was calculated using the height recorded on admission using a wall mounted stadiometer.

Change in body composition was assessed by skinfold measurements using callipers (Holtain Ltd, Crymych, UK) and non-dominant hand-grip strength using a dynamometer (Jamar, Sammons Preston Roylan, Bolingbrook, IL, USA), with technique recommend in practice.

### Blood tests

Blood tests monitoring electrolytes (potassium, magnesium, and phosphate), blood glucose level and thiamine were taken at baseline prior to initiating the nutrition intervention. Electrolyte levels were repeated 4–6 h after initiating enteral nutrition, and at least daily for the first week, and twice weekly in week 2 and week 3. Hypokalaemia was defined as a potassium level < 3.2 mmol/L, and hypomagnesemia was defined as a magnesium level < 0.70 mmol/L, as per the hospital reference range. Hypophosphatemia was defined as a phosphate level ≤ 1.0 mmol/L [16, 34]. Blood glucose levels were measured two times per day during the first week and at least weekly in week 2 and week 3. Hypoglycaemia was defined as a blood glucose level < 3.0 mmol/L [35]. The lowest serum potassium, magnesium, phosphate and blood glucose level was recorded for week 1, 2 and 3 and used in the analysis. Thiamine was repeated at week 1, 2 and 3. The hospital normal reference range for thiamine was 67–200 nmol/L.

### Refeeding syndrome

For this study, refeeding syndrome was identified as the occurrence of life threatening complications (delirium, cardiac arrest, and coma) [6], as well as patients who exhibited all three diagnostic criteria involving electrolyte disturbance, acute circulatory fluid overload and organ dysfunction defined by Rio et al. [36].

### Medical records

Electronic medical records were reviewed at least 3 times/week to review nursing observations (e.g. heart rate, blood pressure, temperature) and weekly physical assessment by the treating medical officer (e.g. monitoring for presence of peripheral oedema). The length of hospital stay, admission to ICU, and number of days to reach medical stability, was also reviewed.

### Analysis

Data was collected and analysed using SPSS for Windows Version 26, IBM Corporation. Continuous outcomes were assessed for normality using the Shapiro–Wilk test. Mean, standard deviation, 95% CI, and effect size reported as d_Cohen_ or Partial Eta squared where appropriate, were reported for parametric data. Normally distributed variables were compared between the two treatment groups using independent t-tests for single measures, (e.g. hospital length of stay, age, white cell count on admission, heart rate on admission, % weight loss prior to admission, weight gain at weeks 1, 2 and 3) and two factor (between subject factor: group; within subject factor: time) repeated measures analysis of variance (ANOVA) for variables with multiple time point measures (e.g. weight, BMI, %mBMI, energy (kcal/kg/day), phosphate, magnesium, potassium, glucose, thiamine, handgrip strength, triceps skinfold, bicep skinfold, suprailiac skinfold). Change in phosphate levels between the two treatment groups was further analysed using analysis of covariance (ANCOVA) with %mBMI as a covariate as the literature has reported degree of malnutrition influencing development of refeeding complications such as hypophosphatemia [15].

Median, interquartile range, and effect size reported as d_Cohen_ were reported for variables not normally distributed (e.g. number of days to reach medical stability; energy intake (kcal/day) for oral, nasogastric feed and total; total and oral intake % macronutrient intake (carbohydrate, protein fat); subscapular skinfold), and between group differences were analysed using a Mann Whitney U test, Wilcox matched pair test, and Friedman ANOVA. Binary outcomes (development of hypophosphatemia with degree of malnutrition as a covariate, medical stability on admission, degree of malnutrition, history of purging, electrolyte replacement, and incidence of hypoglycaemia), were compared using a chi-squared test, with odds ratio (OR) and 95% CI reported. A *p* value < 0.05 was required for statistical significance.

## Results

A total of 26 participants were recruited between 01/11/2017 and 31/12/2018, with 2 patients excluded from analysis due to early discharge from treatment within the first week. Twenty-four participants were included in the analysis, all of whom were female with a diagnosis of AN (DSM 5). There was no significant difference at baseline between the lower carbohydrate/high fat feed (n = 14) and standard feed (n = 10) treatment arms in age (17.5 ± 1.3 vs 17.5 ± 0.9 years, *p* = 0.979), history of purging (3/14 vs 4/10, *p* = 0.393), degree of malnutrition using SGA (12/14 vs 6/10 mild-moderately malnourished; 2/14 vs 4/10 severely malnourished, *p* = 0.192) or %mBMI category (6/14 vs 3/10 mild malnutrition; 3/14 vs 6/10 moderate malnutrition; 4/14 vs 0/10 severe malnutrition, *p* = 0.139), % weight loss prior to admission (17.1% ± 7.8 vs 19.7% ± 8.5, *p* = 0.443), medical instability (10/14 vs 8/10, *p* = 1.000), heart rate (57.7 bpm ± 21.5 vs 59.1 bpm ± 16.2, *p* = 0.865), and white cell count (4.9 × 10^9^/L ± 1.5 vs 5.1 × 10^9^/L ± 1.6, *p* = 0.798).

**Fig. 2:**
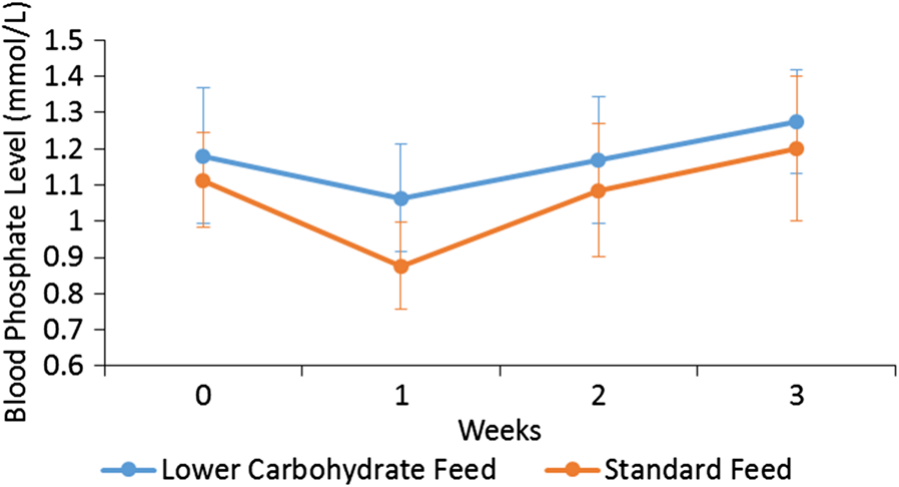
Change in serum phosphate level during 3 weeks of inpatient treatment

During treatment, there was no significant difference between the lower carbohydrate/high fat feed (n = 14) and standard feed (n = 10) treatment arms in the number of days to reach medical stability [median (LQ,UQ) 2.0 (0.0, 3.3) vs 2.0 (0.8, 5.0) days, *p* = 0.512, d_Cohen_ effect size 2.982], phosphate replacement (5/14 vs 6/10, *p* = 0.408, OR 2.70, 95% CI 0.51, 14.37), magnesium replacement (2/14 vs 2/10, *p* = 1.000, OR 1.50, 95% CI 0.17, 12.94), potassium replacement (1/14 vs 2/10, *p* = 0.550, OR 3.25, 95% CI 0.25, 41.91), and hospital length of stay (24.3 ± 11.3 vs 24.4 ± 6.5 days, *p* = 0.975, d_Cohen_ effect size 0.01, 95% CI -0.80, 0.82). A significantly lower rate of hypophosphatemia developed in patients in the lower carbohydrate/high fat feed compared with standard feed treatment arm (5/14 vs 9/10, *p* = 0.013, OR 16.20, 95% CI 1.57, 167.74) during week 1 (Fig. 2). Although degree of malnutrition, defined by %mBMI, is a significant covariate (*p* = 0.018) in the development of hypophosphatemia, it did not affect the outcome as there was no significant association between development of hypophosphatemia and degree of malnutrition (χ^2^ = 1.486, *p* = 0.686). In all 14 patients that developed hypophosphatemia during week 1, this occurred between days 1–5 (mean day 2.9 ± 1.2). There was no significant difference in incidence of hypoglycaemia between the lower carbohydrate/high fat feed and standard feed treatments arms at baseline (1/14 vs 0/10, *p* = 1.000) and at Week 1 (0/14 vs 1/10, *p* = 1.000). No patients developed oedema, clinical refeeding syndrome, or required admission to ICU.

Thiamine levels were measured in only 16 participants at baseline, 11 participants at week 1, and 10 participants at week 2 and 3. There was no significant difference between the lower carbohydrate/high fat feed (n = 9) compared with the standard feed (n = 7) treatment arm at baseline (130.9 nmol/L ± 19.1 vs 147.4 ± 21.0 nmol/L, *p* = 0.122). No significant differences in thiamine levels were found between the two treatment arms during the admission, and all thiamine levels measured at baseline and during treatment were within the normal reference range 67–200 nmol/L.

**Fig. 3:**
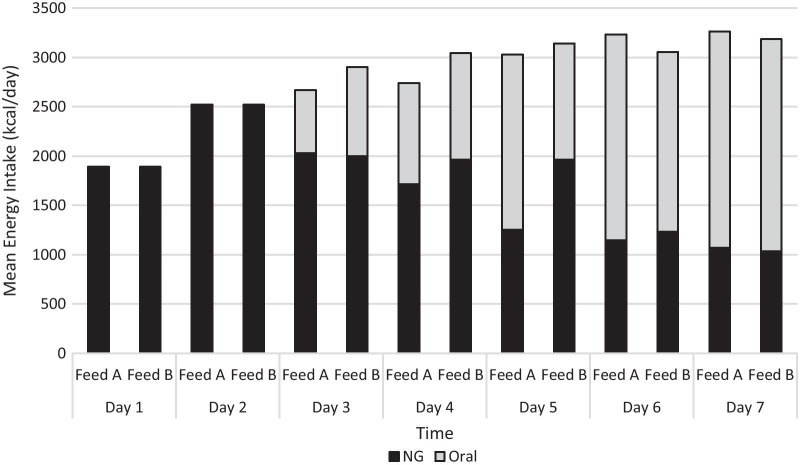
Total Energy intake (kcal) illustrating energy provided from nasogastric enteral feed versus oral meal plan, during first 7 days of admission in participants in lower carbohydrate/high fat feed group (Feed A) and standard feed group (Feed B). No evidence of a difference between the two treatment arms (FEED A, FEED B) in energy intake (kcal) from nasogastric feeds or oral intake (*p* ≥ 0.209)

Additional patient characteristics and changes from baseline to week 1 are presented in Table 1 and Fig. 3. There was no significant difference in weight gain in the lower carbohydrate/high fat feed group compared to the standard feed group at Week 1 (2.7 kg ± 1.9 vs 2.7 kg ± 1.6, *p* = 0.998), Week 2 (4.9 kg ± 1.9 vs 4.6 kg ± 1.5, *p* = 0.669), and Week 3 (6.5 kg ± 2.3 vs 6.4 kg ± 2.0, *p* = 0.959).

**Table 1:** Baseline characteristics and changes after one week of nutritional rehabilitation

	Baseline						Week 1			
	Lower carbohydrate /high fat feed (n = 14)	95% CI	Standard feed (n = 10)	95% CI	Baseline comparison *p* value	Effect size	Lower carbohydrate /high fat feed (n = 14)	95% CI	Standard feed (n = 10)	95% CI
*Anthropometry*										
Body Mass, kg	43.7 ± 4.7	41.0, 46.4	45.1 ± 2.8	43.1, 47.1	0.37	0.35	46.4 ± 5.1	43.5, 49.3	47.9 ± 4.0	45.0, 50.8
BMI, kg/m^2^	16.3 ± 1.7	15.3, 17.3	16.7 ± 0.9	16.1, 17.3	0.53	0.28	17.3 ± 1.8	16.3, 18.3	17.7 ± 1.1	16.9, 18.5
mBMI, %	77.8 ± 9.1	72.5, 83.1	79.3 ± 5.2	75.6, 83.0	0.63	0.19	82.7 ± 9.4	77.3, 88.1	84.0 ± 5.9	79.8, 88.2
Handgrip (L), kg	20.8 ± 3.3	18.9, 22.7	22.7 ± 3.7	20.1, 25.3	0.19	0.55	22.1 ± 4.4	19.6, 24.6	23.1 ± 3.5	20.6, 25.6
Handgrip (R), kg	24.2 ± 4.8	21.4, 27.0	23.2 ± 3.6	20.6, 25.8	0.58	-0.23	25.1 ± 5.0	22.2, 28.0	25.2 ± 3.6	22.6, 27.8
Tricep skinfold, mm	9.5 ± 2.9	7.8, 11.2	9.6 ± 2.7	7.7, 11.5	0.92	0.04	10.5 ± 3.5	8.5, 12.5	10.1 ± 2.5	8.3, 11.9
Bicep skinfold, mm	5.0 ± 1.7	4.0, 6.0	5.1 ± 1.4	4.1, 6.1	0.93	0.06	5.4 ± 2.0	4.2, 6.6	5.3 ± 1.3	4.4, 6.2
Suprailiac skinfold, mm	7.3 ± 2.6	5.8, 8.8	7.0 ± 1.9	5.6, 8.4	0.77	-0.13	8.3 ± 3.1	6.5, 10.1	8.2 ± 2.3	6.6, 9.8
Subscapular skinfold, mm	6.5 [5.6, 8.4]	N/A	6.7 [5.9, 8.4]	N/A	0.48^A^	0.29	6.8 [6.0, 9.3]	N/A	7.5 [6.7, 8.9]	N/A
*Nutrition*										
Total kcal/day (Oral + NG feed)	1890 [1890, 1890]	N/A	1890 [1890, 1890]	N/A	1.00^A^	0.00	3350 [3188, 3350]	N/A	3325 [2844, 3350]	N/A
Kcal/kg/day	43.7 ± 4.8	40.9, 46.5	42.0 ± 2.5	40.2, 43.8	0.28	-0.42	71.1 ± 9.9	65.4, 76.8	66.8 ± 7.5	61.4, 72.2
Energy from Carbohydrate, %	28.3 [28.3, 28.3]	N/A	54.4 [54.4, 54.4]	N/A	< 0.001^A^	3.06	44.8 [44.2, 45.3]	N/A	53.0 [52.9, 56.1]	N/A
Energy from Protein, %	16.8 [16.8, 16.8]	N/A	16.8 [16.8, 16.8]	N/A	1.00^A^	0.00	14.5 [14.4, 14.5]	N/A	14.5 [14.1, 14.5]	N/A
Energy from Fat, %	55.8 [55.8, 55.8]	N/A	29.4 [29.4, 29.4]	N/A	< 0.001^A^	3.06	40.4 [39.8, 40.4]	N/A	32.2 [28.4, 32.3]	N/A
kcal/day from oral intake	0 [0,0]	N/A	0 [0,0]	N/A	1.00^A^	0.00	2300 [1800, 2300]	N/A	2300 [1800, 2300]	N/A
Energy from Carbohydrate, %	0 [0,0]	N/A	0 [0,0]	N/A	1.00^A^	0.00	52.3 [52.3, 57.4]	N/A	52.3 [52.3, 57.4]	N/A
Energy from Protein, %	0 [0,0]	N/A	0 [0,0]	N/A	1.00^A^	0.00	13.4 [12.6, 13.4]	N/A	13.4 [12.6, 13.4]	N/A
Energy from Fat, %	0 [0,0]	N/A	0 [0,0]	N/A	1.00^A^	0.00	33.4 [27.6, 33.4]	N/A	33.4 [27.6, 33.4]	N/A
*Biochemical parameters*										
Phosphate, mmol/L	1.18 ± 0.19	1.07, 1.29	1.11 ± 0.13	1.02, 1.20	0.35	-0.42	1.06 ± 0.15 *	0.97, 1.15	0.88 ± 0.12 *†	0.79, 0.97
Magnesium, mmol/L	0.94 ± 0.09	0.89, 0.99	0.94 ± 0.05	0.90, 0.98	0.85	0.00	0.86 ± 0.08	0.81, 0.91	0.85 ± 0.05	0.81, 0.89
Potassium, mmol/L	3.75 ± 0.44	3.50, 4.00	3.72 ± 0.32	3.49, 3.95	0.84	-0.08	3.67 ± 0.23	3.54, 3.80	3.59 ± 0.10	3.52, 3.66
Glucose, mmol/L	4.8 ± 0.9	4.3, 5.3	5.5 ± 1.2	4.6, 6.4	0.22	0.68	4.4 ± 0.7	4.0, 4.8	4.5 ± 0.8	3.9, 5.1

	ANOVA Results					
	Time		Feed		Interaction	
	*p* value	Effect size	*p* value	Effect size	*p* value	Effect size
*Anthropometry*						
Body Mass, kg	< 0.001	0.71	0.428	0.03	0.998	0.00
BMI, kg/m^2^	< 0.001	0.72	0.566	0.02	0.978	0.00
mBMI, %	< 0.001	0.72	0.670	0.01	0.931	0.00
Handgrip (L), kg	0.059	0.15	0.346	0.04	0.289	0.05
Handgrip (R), kg	0.006	0.30	0.790	0.00	0.274	0.05
Tricep skinfold, mm	0.001	0.43	0.935	0.00	0.241	0.06
Bicep skinfold, mm	0.001	0.41	0.954	0.00	0.214	0.07
Suprailiac skinfold, mm	< 0.001	0.60	0.857	0.00	0.616	0.01
Subscapular skinfold, mm	0.395^B^	0.35	0.002^C^	2.87	0.005^D^	3.96
*Nutrition*						
Total kcal/day (Oral + NG feed)	0.599^B^	0.20	0.001^C^	4.00	0.005^D^	2.30
Kcal/kg/day	< 0.001	0.94	0.248	0.06	0.365	0.04
Energy from Carbohydrate, %	< 0.001^B^	3.06	0.001^C^	4.00	0.797^D^	0.16
Energy from Protein, %	0.478^B^	0.25	0.001^C^	4.67	0.004^D^	4.11
Energy from Fat, %	< 0.001^B^	3.06	0.001^C^	4.67	0.027^D^	1.95
kcal/day from oral intake	0.841^B^	0.07	0.001^C^	4.08	0.004^D^	2.49
Energy from Carbohydrate, %	0.841^B^	0.07	0.001^C^	4.08	0.004^D^	2.49
Energy from Protein, %	0.841^B^	0.07	0.001^C^	4.08	0.004^D^	2.49
Energy from Fat, %	0.841^B^	0.07	0.001^C^	4.08	0.004^D^	2.49
*Biochemical parameters*						
Phosphate, mmol/L	0.086	0.13	0.013	0.26	0.035	0.20
Magnesium, mmol/L	< 0.001	0.71	0.853	0.00	0.396	0.03
Potassium, mmol/L	0.193	0.08	0.584	0.01	0.779	0.00
Glucose, mmol/L	0.058	0.21	0.141	0.13	0.994	0.00

Data are presented as means ± standard deviations when normally distributed and medians [interquartile range] when not normally distributed

*Significant difference to baseline based on ANCOVA Bonferonni post hoc results and using percent median BMI on admission as a covariate

^†^Signficant difference to Lower carbohydrate/high fat feed at same time point on ANCOVA Bonferonni post hoc results and using percent median BMI on admission as a covariate

^A^Mann Whitney U test for lower carbohydrate/high fat feed versus standard feed at Baseline

^B^Mann Whitney U test for lower carbohydrate/high fat feed versus standard feed at Week 1

^C^Wilcoxon matched paired test for lower carbohydrate/high fat feed Baseline versus Week 1

^D^Wilcoxon matched paired test for standard feed Baseline versus Week 1

At an interim point review, the trial was ceased, based on the significant finding of the primary outcome measure of development of hypophosphatemia reduced in the lower carbohydrate/high fat feed compared with the standard feed treatment arm at the interim analysis. No other significant differences were found after Week 1 in any of the variables analysed (Additional file 2: Table S2). There were no adverse or unintended outcomes reported in either treatment group.

## Discussion

This is the first study to compare refeeding outcomes for malnourished adolescent and young adult patients given isocaloric nutritional therapy which was either high (54%) or low (28%) carbohydrate enteral feeds. Our results show a lower carbohydrate/high fat enteral feed in adolescent and young adult patients hospitalised with AN resulted in lower levels of hypophosphatemia compared with those provided with a standard higher carbohydrate feed. No other significant differences were found between the two treatment arms during the first 3 weeks of admission.

Providing < 40% carbohydrate to refeed patients with eating disorders has been recommended [13]. Study outcomes of refeeding using nasogastric feeding tubes above current international recommendations (exceeding 1200 kcal/day), providing 44–46% [10, 22] or ≤ 50% carbohydrate [18] have been reported. Mathews et al. [10] reported no significant differences in rates of hypophosphatemia during the first 10 days of admission, in a retrospective pre-test-post-test study comparing a low-calorie (LC) protocol (1000 kcal, 45–55% carbohydrate, 20% protein, 30–35% fat) to a higher-calorie (HC) protocol (1500 kcal, 46% carbohydrate, 20% protein, 34% fat), in 119 medically compromised adult patients with eating disorders. The highest rate of hypophosphatemia (defined as below normal reference range 0.81–1.45 mmol/L) was on admission (23% LC vs 12% HC, *p* = 0.196), followed by Day 1 (19% LC vs 12% HC, *p* = 0.307). The LC group (n = 26) were provided with an oral based meal program, with nasogastric feeding provided only if oral feeding was deemed unsuccessful, whereas the HC group (n = 93) utilised nasogastric feeding within 24 h of admission. The authors did report a higher incidence of hypoglycaemia (defined as serum glucose level < 3.0 mmol/L or skin prick glucose level < 4.0 mmol/L) in the LC group compared to the HC group (31% vs 10%, *p* = 0.012), and suggested the use of continuous nasogastric feeding with constant supply of carbohydrate in the HC group may have contributed to the reduced incidence of hypoglycaemia. In the current study, both treatment arms reported a higher incidence of hypophosphatemia, compared with Mathews et al. [10], in the lower carbohydrate/high fat feed and the standard feed (35.7% vs 90%, *p* = 0.013). However, in addition to starting patients on a higher calorie intake (1890 kcal/day), hypophosphatemia was defined as PO_4_ ≤ 1.0 mmol/L in the current study rather than PO_4_ < 0.81 mmol/L. Furthermore, there were no significant differences between treatment arms in blood glucose levels at baseline and week 1. The use of initial continuous nasogastric feeding in both treatment arms in the current study combined with the low rates of hypoglycaemia observed, further support the suggestion by Kohn et al. [13] and the results of Mathews et al. [10], that a constant supply of carbohydrate through the use of continuous NG feeds may reduce the rate of hypoglycaemia in this patient population.

Other studies that reported outcomes on patients hospitalised with AN provided with enteral feeds, include Madden et al. [18] providing 2400 kcal/day, limiting carbohydrate to ≤ 50% energy; and Agostino et al. [22] providing 1617 kcal/day, 44% carbohydrate to adolescent patients. However, while both studies report 0% hypophosphatemia in patients receiving NG feeds, these results are not comparable to the current study as both studies prescribed prophylactic phosphate supplementation prior to nutrition intervention which would mask the development of hypophosphatemia.

Our results suggest the use of an enteral feed providing only 28% energy from carbohydrate reduced the incidence of hypophosphatemia observed compared with a standard feed. Both treatment arms in this study received a higher calorie protocol providing patients with 1890 kcal on admission. Electrolyte disturbances were treated with oral supplementation, and no cases of clinical refeeding syndrome or admissions to ICU occurred. These results support higher caloric prescription than recommendations in current guidelines. The use of a lower carbohydrate/high fat enteral feed provides a further option for clinicians to consider when treating patients hospitalised with AN, rather than providing low energy intakes and risking an underfeeding syndrome and even weight loss in this already malnourished and often medically compromised patient population. There were no significant differences in any variables observed between the two treatment arms after week 1, and this may be explained by the increase in oral intake and reduction of calories provided through nasogastric tube feeding after week 1, thereby reducing the difference in macronutrient composition received by the two treatment arms in Week 2 and 3.

Several limitations affect the generalisability and interpretation of results. The sample size of this study and the effect size observed were both small. The small sample size was attributed to the trial being ceased early following the interim analysis identifying a significant difference between the two treatment arms in the primary outcome measure of incidence of hypophosphatemia. The age range in the inclusion criteria prohibits the findings of this study being applicable to older patients who may also have a more severe and enduring course of illness. Furthermore, while all patients were diagnosed as malnourished using a validated nutrition assessment tool, the admission BMI or %mBMI categorised the majority of patients as mild to moderately malnourished therefore limiting the validity of results in severely malnourished patients who are at the highest risk of developing refeeding complications.

The strengths of the study are that it is the first of its kind to compare two different feeding formulas with different macronutrient compositions, in patients hospitalised with AN using a double-blinded randomised controlled trial design with controls for bias, thereby enhancing reliability of study findings. Furthermore, by focusing on the adolescent and young adult population hospitalised with AN, the study addresses the urgent need to identify effective treatments to reduce length of hospital stay and associated costs, with the hope of preventing severe and enduring course of illness in the longer term.

Further research is required to assess the safety and efficacy of the use of a lower carbohydrate/high fat enteral feed in more severely malnourished and older patients with AN, as well as other patient groups considered at risk of developing refeeding complications, specifically patients with a history of prolonged fasting or low energy intake [37]. A comparison of patient outcomes in higher energy oral based feeding protocols versus enteral feeding protocols manipulating macronutrient content is also warranted to examine the safety, feasibility and discomfort that may be experienced by patients in either treatment model.

Furthermore, while the current study did not report a significant difference in weight gain between the two treatment arms, future studies incorporating indirect calorimetry measurements into the study design are recommended. This will help confirm if patients fed a high carbohydrate/low fat diet have a higher basal metabolic rate due to increased diet-induced thermogenesis, compared to patients fed a low carbohydrate/high fat diet, as reported by Russell et al. [38].

## Conclusion

The results of this study indicate that enteral nutrition provided to hospitalised malnourished young people with AN using a lower carbohydrate/high fat formula (28% carbohydrate, 56% fat) seems to provide protection from hypophosphatemia in the first week compared to when using a standard enteral formula. Further research is required to confirm this finding in more severely malnourished and older patients with AN, as well as other malnourished populations with a history of prolonged fasting or low energy intake.

## Supplementary Information

**Additional file 1**: **Table S1**. Macronutrient composition of standardised meal plans.

**Additional file 1**: **Table S2**. Changes at Week 2 and Week 3 of nutritional rehabilitation.

## Data Availability

The datasets used and/or analysed during the current study are available from the corresponding author on reasonable request.

## Abbreviations

AN: Anorexia nervosa
ANCOVA: Repeated measures analysis of covariance
ANOVA: Repeated measures analysis of variance
APA: American Psychiatric Association
ASPEN: American Society for Parenteral and Enteral Nutrition
BMI: Body mass index
%mBMI: Percent median body mass index
HC: High-calorie
LC: Lower-calorie
MARSIPAN: Management of really sick patients with anorexia nervosa
NICE: National Institute for Health and Care Excellence
OR: Odds ratio
