# Supplementary material for: A standard enteral formula versus an iso-caloric lower carbohydrate/high fat enteral formula in the hospital management of adolescent and young adults admitted with anorexia nervosa: a randomised controlled trial
Source: J Eat Disord. 2021 Dec 11;9:160. doi: 10.1186/s40337-021-00513-6 (PMC8666027; doi:10.1186/s40337-021-00513-6)
Supplement: Supplementary file 1 — Additional file 1: Table S1. Macronutrient composition of standardised meal plans. [file 40337_2021_513_MOESM1_ESM.docx]

**Supplement Table 1.** Macronutrient composition of standardised meal plans

| **Energy Content** | **% Carbohydrate** | **% Protein** | **% Fat** |
| --- | --- | --- | --- |
| 1800 kcal | 57.4 | 12.6 | 30.0 |
| 2300 kcal | 52.3 | 13.4 | 33.4 |
| 2800 kcal | 47.9 | 13.9 | 37.4 |
| 3300 kcal | 46.6 | 15.3 | 37.5 |
| 3800 kcal | 49.1 | 14.8 | 35.3 |
